# Validation of Functional Connectivity of Engineered Neuromuscular Junction With Recombinant Monosynaptic Pseudotyped ΔG-Rabies Virus Tracing

**DOI:** 10.3389/fnint.2022.855071

**Published:** 2022-05-20

**Authors:** Ulrich Stefan Bauer, Vegard Fiskum, Rajeevkumar Raveendran Nair, Rosanne van de Wijdeven, Clifford Kentros, Ioanna Sandvig, Axel Sandvig

**Affiliations:** ^1^Department of Neuromedicine and Movement Science, Norwegian University of Science and Technology (NTNU), Trondheim, Norway; ^2^Centre for Neural Computation, Kavli Institute for Systems Neuroscience, Norwegian University of Science and Technology (NTNU), Trondheim, Norway; ^3^Department of Cancer Research and Molecular Medicine, Norwegian University of Science and Technology (NTNU), Trondheim, Norway; ^4^Institute of Neuroscience, University of Oregon, Eugene, OR, United States; ^5^Department of Clinical Neuroscience, Umeå University Hospital, Umeå, Sweden; ^6^Department of Community Medicine and Rehabilitation, Umeå University, Umeå, Sweden

**Keywords:** amyotrophic lateral sclerosis (ALS), motor neurons (MNs), pseudo-organoids, neuroengineering, *in vitro* modeling

## Abstract

Current preclinical models of neurodegenerative disease, such as amyotrophic lateral sclerosis (ALS), can significantly benefit from *in vitro* neuroengineering approaches that enable the selective study and manipulation of neurons, networks, and functional units of interest. Custom-designed compartmentalized microfluidic culture systems enable the co-culture of different relevant cell types in interconnected but fluidically isolated microenvironments. Such systems can thus be applied for ALS disease modeling, as they enable the recapitulation and study of neuromuscular junctions (NMJ) through co-culturing of motor neurons and muscle cells in separate, but interconnected compartments. These *in vitro* systems are particularly relevant for investigations of mechanistic aspects of the ALS pathological cascade in engineered NMJ, as progressive loss of NMJ functionality may constitute one of the hallmarks of disease related pathology at early onset, in line with the dying back hypothesis. In such models, ability to test whether motor neuron degeneration in ALS starts at the nerve terminal or at the NMJ and retrogradely progresses to the motor neuron cell body largely relies on robust methods for verification of engineered NMJ functionality. In this study, we demonstrate the functionality of engineered NMJs within a microfluidic chip with a differentially perturbable microenvironment using a designer pseudotyped ΔG-rabies virus for retrograde monosynaptic tracing.

## Introduction

Amyotrophic Lateral Sclerosis (ALS) is a progressive, fatal neurodegenerative disease that primarily affects upper and lower motor neurons (MN) with a lifetime risk of 1:350 in men and 1:400 in women, making it the third most prevalent neurodegenerative disease (Johnston et al., 2006; Hirtz et al., 2007). The progressive degeneration and, ultimately, loss of motor neurons affects synaptic transmission at the neuromuscular junction (NMJ). As a result, early symptoms of the disease can manifest as muscle twitches or cramps and gradually progress to include spasticity, leading to muscle atrophy, loss of control of voluntary movements, and inevitably respiratory failure, typically within 3–5 years after symptom onset (Nardo et al., 2016; De Vos and Hafezparast, 2017). The critical role and the potentially selective NMJ vulnerability to early onset ALS pathology before progressive retrograde motor neuron degeneration and pathological spread within interconnected networks constitutes the dying back hypothesis (as opposed to the dying forward hypothesis), and has been evidenced both in clinical studies and in animal models of ALS (Baker, 2014; Cappello and Francolini, 2017; Campanari et al., 2019; Tsitkanou et al., 2019). While recent findings have demonstrated the importance of dysfunction of brain networks in ALS (Renga, 2022), the neuromuscular junction remains a critical site in the disease, which requires appropriate tools to investigate in detail.

A wide range of mutations have been identified as causing the familial form of the disease, but while our knowledge concerning underlying mutations has been rapidly expanding in recent years, our understanding of the molecular pathology, particularly the onset, and mechanism by which the respective mutations cause the disease, remains lackluster. Similarly, the mechanisms underlying the sporadic form of the disease are poorly understood.

Current preclinical *in vivo* models of ALS make use of transgenic mice overexpressing some of the most common gene mutations associated with ALS, typically SOD1, TDP-43, FUS, or C9ORF72. Each model has distinctive advantages, however, no single model faithfully mimics disease complexity (Kabashi et al., 2008; O’Rourke et al., 2015; Philips and Rothstein, 2015; Nardo et al., 2016; Sharma et al., 2016). An alternative or complementary approach to *in vivo* ALS models, is presented by *in vitro* cell-based models. In the last decade, such *in vitro* models have undergone significant development, especially after the advent of cell reprogramming technologies enabling the conversion of somatic cells into induced pluripotent stem cells (IPSCs) after transduction with only four genes (Oct4, Sox2, Klf4, and c-Myc) (Takahashi and Yamanaka, 2006). Furthermore, iPSCs can be differentiated into specific neuronal subtypes, including MN (Amoroso et al., 2013; Richner et al., 2015; Abernathy et al., 2017).

Recent developments in *in vitro* modeling platforms, such as microelectrode arrays (MEAs) and microfluidic chips provide new options for investigating the electrophysiological behavior of neural networks (van de Wijdeven et al., 2018, 2019). In the context of ALS, of particular relevance are models based on the recapitulation of the NMJ in healthy and perturbed conditions. Indeed, a number of studies have demonstrated the applicability of various bioengineering methods for creating muscle-neuron co-cultures, including functional NMJ *in vitro* (Morimoto et al., 2013; Park et al., 2013; Demestre et al., 2015; Ionescu et al., 2016; Happe et al., 2017; Lewandowska et al., 2018; Mills et al., 2019). In the present study, we model functional human NMJs by compartmentalized culture of iPSC-derived MN pseudo-organoids and myotubes in a differentially perturbable multi-nodal microfluidic chip and demonstrate that NMJ function can be validated through monosynaptic retrograde tracing using a designer pseudotyped ΔG-rabies virus (RV).

## Materials and Methods

### Cell Culture

Induced pluripotent stem cells (iPSCs) were purchased from Takara Bio Europe AB (Y00305). The cells were expanded on Laminin 521 (LN521, BioLamina) coated flasks using iPS expansion media containing Knockout-DMEM/F-12 (12660012, Invitrogen), 20% Xeno-free CTS Knockout Serum Replacement (A1099202, Thermo Fisher Scientific), Penicillin-streptomycin, Non-essential amino acids, L-glutamine, 2-Mercaptoethanol 55 mM (21985-023, Thermo Fisher Scientific), and 4 ng/ml FGF-Basic (AA 10-155) Recombinant Human Protein (PHG0026, Life Technologies).

A modified version of the protocol published by Amoroso et al. (2013) was used to differentiate iPS cells to motor neurons. iPSC-derived motor neurons were maintained in motor neuron media (MN media) consisting of Neurobasal Medium (21103049, Thermo Fisher Scientific), N2 supplement (17502-048, Gibco), B27 supplement (17504-044, Gibco), Non-essential amino acids, L-glutamine, Penicillin-streptomycin, 0.4 μg/ml Ascorbic acid, 25 μM L-glutamate, 1 μM all-trans Retinoic acid, 25 μM 2-Mercaptoethanol, 20 μM Y-27632 (Y0503, Sigma-Aldrich).

Primary human skeletal myoblasts (A11440, Thermo Fisher Scientific) were expanded in DMEM + L-glutamine (21041-025, Gibco), 20% Embryonic stem-cell FBS (16141061, Gibco), 40 ng/ml Dexamethasone (D2915, Sigma-Aldrich), 20 μg/ml Insulin (I-2643, Sigma-Aldrich), 20 ng/ml EGF Recombinant Human Protein (10605HNAE250, Life Technologies), 20 ng/ml FGF-Basic (AA 10-155) Recombinant Human Protein, 1:100 Penicillin-streptomycin.

Myoblast to myotube differentiation was achieved by switching to myoblast differentiation media (MDM) DMEM basal medium (11885-084, Thermo Fisher Scientific), 2% Horse Serum (16050-130, Thermo Fisher Scientific), 1:100 Penicillin-streptomycin.

### Induced Pluripotent Stem Cells Derived Motor Neuron Cultures on Microelectrode Arrays

Five 60EcoMEA-Glass microelectrode arrays (Multichannel systems) were disinfected with 70% ethanol for 5 min or less and washed with PBS before sterilizing under ultraviolet light overnight. The MEAs were then treated with fetal bovine serum (FBS) overnight to ensure the surfaces were hydrophilic. After removing the FBS, the MEAs were coated with Matrigel (Sigma-Aldrich), diluted 1:30 in cell expansion media. Each MEA was coated with 750 μL of diluted Matrigel and left for 30 min at 40°C, followed by 30 min at room temperature. After removing the coating, the MEAs were seeded with approximately 20,000 rat astrocytes at a concentration of 100,000 cells/mL. Three days after seeding the astrocytes, approximately 100,000 MNs were seeded onto each MEA at a concentration of 1,750,000 cells/mL. Culture media was replaced 3 times per week up until culture age of 28 days, after which media was replaced once per week.

### *In vitro* Electrophysiology and Antigenic Profile of Motor Neuron Networks

Recordings of electrical activity of the motor neuron networks were obtained on day 8, 15, 22, 28, 35, 42, 49, 56, 63, and 70 days *in vitro* (DIV). Each recording was 4 min long at 10,000 Hz and was made at least 24 h after media replacement. Only baseline activity was recorded with no stimulation. At 8 DIV, networks showed very little or no activity, and this data was not considered in further analysis. Up until 42 DIV, *N* = 5 networks, and between 42 and 70 DIV, *N* = 4 networks. Spike detection was performed in NeuroExplorer 5, after filtering the recorded signal through a fourth order Butterworth bandpass filter from 300 to 3,000 Hz, and spikes were identified in signals that were > 7 *SD* from the mean signal, and the timestamps of spikes were recorded. These spike trains were exported to MatLab R2019a (MathWorks) for further analysis.

Electrophysiological activity measures were assessed across the whole networks, using the entire MEA. Firing rate was established by dividing total number of recorded spikes during the recording across all electrodes by the length of the recording. Network bursts were identified by examining a firing rate histogram using 50 ms time bins. Each bin which exceeded a threshold of the mean bin firing rate plus 5 standard deviations were initially considered to be bins with network bursts. These initial bursts were then pruned by removing bins where fewer than 20% of the active electrodes on the MEA were active. Finally, adjacent bins were combined to single, longer bursts.

In order to confirm the identity of the cells as MNs, cells were cultured in parallel to the MEAs in 4 wells on an 8-well Ibidi chip. At 53 DIV, these were stained for motor neuron markers. First, the cell media was aspirated, and the cells were washed with warm PBS. Then they were fixed with 4% paraformaldehyde for 15–25 min before being treated with a block solution of 5% goat serum and 0.3% Triton-X in PBS for 1–2 h. After removing the block, three wells were stained with primary antibodies for Islet 1 (Abcam, ab86472, 1:250 dilution), HB9 (Abcam, ab221884, 1:2,500 dilution) and heavy neurofilament (Abcam, ab4680, 1:1,000 dilution) with 1% goat serum and 0.1% Triton-X in PBS, while the last well was used as a control, filled only with PBS with goat serum and Triton-X. The primary antibody was left overnight at 4°C. The next day, the wells were washed with PBS 3 times 10 min, before adding secondary antibodies (Thermo Fisher Scientific, A11001, A11036, A21449, all at 1:1,000 dilution) with 1% goat serum and 0.1% Triton-X in PBS to 3 out of the 4 wells, including to the well which was not stained with the primary antibodies. The last well was filled with only PBS with goat serum and Triton-X, so that there was a control with primary antibody only and one with secondary antibody only. The wells were covered in aluminum foil to prevent light exposure and left for 2 h, and Hoechst staining added (Sigma-Aldrich 14533, 20 μg/mL at 1:10,000 dilution) during the last 5 min. After removing the secondary antibodies, the wells were washed 3 times 15 min before covering the cells with a glass coverslip using Fluoroshield Mounting Medium (Abcam ab104135). The finished slides were then left at 4°C overnight. When imaging the cells, exposure time was set to 300–400 ms for all stains except Hoechst, which was set to 8–15 ms.

### Microfluidic Chip Design and Production

In this work, we used an in-house developed semi-open system microfluidic chip comprising four cell compartments interconnected through micro-sized tunnels only permissible to axons. In a recent publication, we demonstrated the compatibility of such a microfluidic chip with neuronal aggregates (van de Wijdeven et al., 2018). A photoresist mold was fabricated using standard lithographic techniques, more details regarding the design and the fabrication procedure can be found in a previous study (van de Wijdeven et al., 2018). Poly(dimethylsiloxane) (PDMS) was cast on top of the mold and cured in an over at 65°C for 4 h. Afterward, the PDMS was carefully peeled from the mold and the cell compartments were opened using a punching device (Ø 6 mm). The PDMS debris was removed with adhesive tape and consecutive washes in acetone, ethanol (70%) and deionized DI water. The chips were semi-dried with an airgun and left to dry overnight. Subsequently, the chips were irreversibly bonded onto glass coverslips (24 × 32 mm Menzel-Gläser) by exposing both surfaces to oxygen plasma for 1 min followed by directly heating the assembled device at 75°C for 30 s. Finally, the chips were filled with DI water and kept at 4°C for storage. Prior to coating, the chips were sterilized under UV light.

### Microfluidic Chip Seeding and Maintenance

Prior to seeding, chips were coated with Poly-L-Ornithine (PLO) solution (P4957, Sigma) and Natural Mouse (NM) Laminin (23017-015, Gibco). The sterile water the chips were stored with was removed and replaced with PLO solution. Using hydrostatic pressure within the chip, the channels were flushed through with PLO for 30 min before the PLO solution was removed and replaced with fresh PLO solution. The chips were placed in a humidified incubator for at least 2 h at 37°C, 5% CO_2_ before being washed 3x using cell culture-grade distilled water, allowing the channels to be flushed through by hydrostatic pressure for 30 min for each wash. The final wash was replaced with 16.5 μg/mL NM Laminin in L-15-Medium (L5520, Sigma) supplemented with 1.9 mg/ml sodium bicarbonate, the channels were again flushed through for 30 min by hydrostatic pressure before the NM Laminin solution was removed and replaced with fresh NM Laminin solution. The chips were placed in a humidified incubator for at least 2 h 37°C, 5% CO_2_. Before seeding, the outer compartments were filled with MN media and the central compartment with MDM. MN aggregates were selected for a diameter of around 1 mm and placed in the compartments by careful aspiration with a P1000 pipette. The aggregates were positioned close to the active zone using a P50 pipette tip. The central compartment was seeded with 35,000 myoblasts. After seeding media levels were adjusted to ensure a slight hydrostatic pressure gradient from the outer to the inner compartment all chips were incubated in parafilm-sealed petri dishes alongside reservoirs containing cell couture-grade distilled water in humidified incubators at 37°C, 5% CO_2_. 3/4 Media changes were carried out every 48 h.

### Immunocytochemistry of *in vitro* Neuromuscular Junctions

Cells were fixed in 4% Parafomaldehyde in PBS, blocking was carried out with 5% Goat serum and 0.6% Triton-X in PBS and primary and secondary immunostaining in 2.5% Goat serum and 0.3% Triton-X in PBS (for first BTX staining and staining after the non-blinded BTX activity abolishing experiment 0.06 and 0.03% Triton-X were used). Immunostainings were carried out using 1:50 mouse anti-Synaptophysin (ab8049, abcam), 1:100 rabbit anti-Troponin I (701585, Thermo Fisher Scientific), 1:200 mouse anti-Troponin T (SAB420717, Sigma-Aldrich), 1:50 Mouse anti-Ryanodine Receptor (ab2827, abcam), 1:200 rabbit anti-Calcium channel L type DHPR alpha 2 subunit/CACNA2D1 (ab238110, abcam) 1:5,000 chicken Neurofilament-Heavy (ab4680, abcam), 1:100 rabbit anti-Nicotinic Acetylcholine Receptor alpha 1 (ab221868, abcam), 1:2,500 chicken anti-Beta III Tubulin (ab41489, abcam), 1:50 rabbit anti-HB9 (ab221884, abcam), 1:50 rabbit anti-Islet1 (ab109517, abcam), 1:1,000 mouse anti-NeuN (ab104224, abcam), 1:20 rabbit anti-Galactocerebroside (AB142, Sigma-Aldrich), 1:1,000 mouse anti-Beta III Tubulin (ab119100, abcam), and 1:5,000 chicken anti-Glial fibrillary acidic protein (ab4674, abcam). Nuclear staining was carried out using Hoechst DNA stain (bisBenzimide H 33342 trihydrochloride, 14533, Sigma-Aldrich).

Secondary antibody staining was carried out using 1:500 Alexa Fluor 488 goat anti-mouse IgG antibody (A-11001, Life Technologies), 1:500 Alexa Fluor 488 goat anti-rabbit (A-11008, Life Technologies), 1:500 Alexa Fluor 568 goat anti-mouse IgG antibody (A-11019, Life Technologies), 1:500 Alexa Fluor 568 goat anti-rabbit IgG antibody (A-11079, Life Technologies), 1:500 Alexa Fluor 647 goat anti-rabbit (A-21244, Life Technologies), and 1:500 Alexa Fluor 647 goat anti-chicken (A-21449, Life Technologies).

Alpha-Bungarotoxin (α-BTX) labeling was carried out simultaneously with secondary antibody labeling. A 1 mg/ml stock of Alexa Fluor™ 488 conjugate of α-Bungarotoxin (B13422, Thermo Fisher Scientific) was used at 1:100 dilution in the secondary antibody solution.

### Alpha-Bungarotoxin Functional Assay

Chips seeded as described above were allowed to mature for 21 days after seeding. Chips were selected for high contractile activity of myotubes that had visual contact with axons (more than 1 contraction per minute over 5 min with at least 1 contraction in each minute, the day prior to the experiment). Video of pre-experiment activity was recorded for 1 min followed by counting of the total number of contractions in 10 min. The cells were allowed to recover for 1 h at 37°C, 5% CO_2_ before addition of 1:100 of α-BTX at a final concentration of 1.25 μM or of 0.75 μl sterile PBS and incubation of 10 min at 37°C, 5% CO_2_. The α-BTX was only added to the central compartment containing the myotubes, which was fluidically isolated by hydrostatic pressure throughout the incubation. Another 1 min video of activity after intervention was recorded before counting the total number of contractions in 10 min. Cells were fixed immediately after the final count. For quantitative analysis the experiment was repeated with 10 min video being recorded as baseline followed by 1 h recovery at 37°C, 5% CO_2_ and treatment. Another 10 min video was recorded after treatment, and blinded quantification was carried out for both.

### EnvA Pseudotyped-G Deleted Rabies Virus (ΔG-RV) Production

Endotoxin free plasmid maxipreps (Qiagen) were made for all transfections. pAAV-CMV-TVAmCherry-2A-oG (#104330), and pCAG-YTB (#26721) for expressing TVA and Rabies Glycoprotein were purchased from Addgene.

EnvA pseudotyped-G deleted rabies virus expressing GFP (EnvA-ΔG-RV-GFP) or mCherry (EnvA-ΔG-RV-mCherry) transgene were produced as described in Wickersham et al. (2010) and Osakada and Callaway (2013). Briefly, for the recovery of ΔG-RV, 293T cells were co-transfected with the rabies genome construct cSPBN-4GFP (Addgene #52487) along with helper plasmids pcDNA-SADB19N (#32630), pcDNA-SADB19P (#32631), pcDNASADB19L (#32632), pcDNA-SADB19G (#32633), and pCAGGS T7 (#59926). Transfection was carried out using Lipofectamine 2000 (Thermo Fisher Scientific). The transfected 293T cells were maintained in 10% FBS/DMEM in a humidified atmosphere of 5% CO_2_ at 37°C. ΔG-RV particles rescued from the cDNA were amplified by passaging for several days on a complementing cell line BHK-B19G2 cells. For pseudotyping with EnvA, BHK-EnvA cells were infected with unpseudotyped ΔG-RV at a multiplicity of infection (MOI) of 0.5. Twenty-four hours post-infection, infected cells were washed with DPBS, dissociated with 0.25% trypsin-EDTA and reseeded on multiple 15 cm dishes in a humidified atmosphere of 3% CO_2_ at 35°C as described. Incubation medium was harvested 48 h later, filtered using 0.45 μm sterile filter and viral particles were concentrated by ultracentrifugation at approximately 50,000 g for 2 h at 4°C, for producing high titer EnvA-pseudotyped ΔG-RV. The pellets were resuspended in DPBS and concentrated using Amicon Ultra centrifugal filters (Millipore). Unpseudotyped ΔG-RV and EnvA-pseudotyped ΔG-RV were titrated by serial dilutions using HEK293T cells and HEK293-TVA800 cells, respectively. Titer of viral stocks EnvA-ΔG-RV-GFP and EnvA-ΔG-RV-mCherry were determined as approximately 1,010 infectious particles/ml. The pseudotyped ΔG-rabies virus was produced by the Viral Vector Core Facility, Kavli Institute for Systems Neuroscience, NTNU, Norway.

### Retrograde Tracing With Rabies Virus *in vitro*

The same overall layout in the microfluidic chips as in previous experiments was used. The myoblasts were expanded and nucleofected at P2 using P5 Primary Cell 4D-Nucleofector^®^ X Kit L (Lonza, V4XP-5012) with the pCAG-YTB (26721, Addgene) or pAAV-CMV-TVAmCherry-2A-oG (104330, Addgene) that expresses exogenous receptor TVA and the rabies glycoprotein (B19G or optimized G) or pmaxGFP as control (part of the P5 Primary Cell 4D-Nucleofector^®^ X Kit L, V4XP-5024, Lonza) plasmids using the EY-100 setting in the Lonza 4D-Nucleofector™ Core Unit (AAF-1002B, Lonza) and 4D-Nucleofector™ X Unit (AAF-1002X, Lonza). Cultures were allowed to develop without interference until 15 days *in vitro* (DIV) with full media changes every other day. At 15 DIV, the pAAV-CMV-TVAmCherry-2A-oG-nucleofected or pCAG-YTB-nucleofected myotubes were, respectively, infected with EnvA-ΔG-RV-GFP or EnvA-ΔG-RV-mCherry virus in myotube differentiation media overnight. After incubation, the media were removed and the compartments were washed once with warm MDM and again filled with MDM. Full media changes were carried out every other day thereafter.

pAAV-CMV-TVAmCherry-2A-oG was a gift from Marco Tripodi (Addgene plasmid #104330; RRID:Addgene_104330),^1^ pCAG-YTB was a gift from Edward Callaway (Addgene plasmid #26721; RRID:Addgene_26721).^2^

## Results

### Spontaneous Electrical Activity Profile of Induced Pluripotent Stem Cells Derived Motor Neuron Networks on Microelectrode Arrays

**FIGURE 1:**
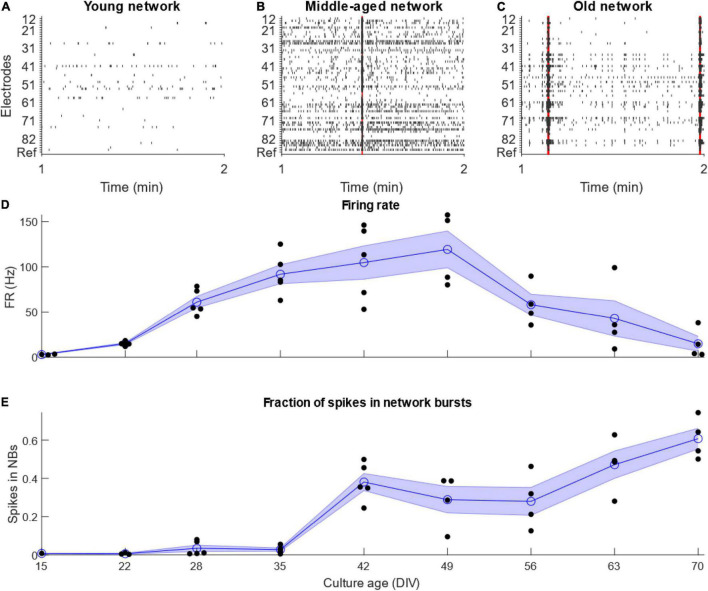
Development of motor neuron activity. Development of activity of networks of IPSC-derived motor neurons. **(A–C)** Example raster plots showing 1 min of representative activity from the different stages of motor neuron network development. Red highlights show network bursts. **(D)** Firing rate increased until 49 DIV, after which it decreased quickly. **(E)** The fraction of total recorded spikes which occurred in network bursts increased for as long as the cultures were maintained. *N* = 5 for culture age 15–42 DIV, *N* = 4 from 49 to 70 DIV. Blue line and shaded region shows mean ± standard error of the mean, while black points show individual MEAs.

Analysis of the data obtained from electrophysiological recordings of iPSC derived MN networks on 60-electrode MEAs revealed the spontaneous electrical activity profile of these networks over time. Based on the firing rate of these MNs across the entire MEA and the fraction of recorded spikes which occurred within network bursts, we could classify the activity of the MN into three categories: (i) “Young” networks, 15–35 DIV characterized by low, but increasing, firing rate and low fraction of bursting; (ii) “Middle-aged” networks, 42–56 DIV showing high firing rate and medium bursting; and (iii) “Old” networks, 63–70 DIV showing low firing rate and high bursting (Figure 1).

**FIGURE 2:**
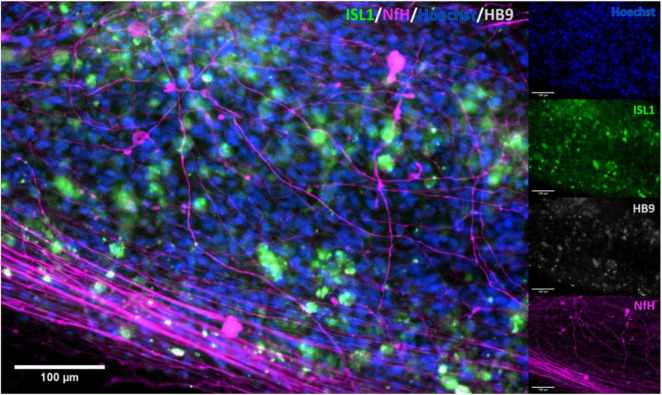
Immunocytochemical stain for motor neuron markers. Staining for motor neuron specific markers clearly showed the presence of these in the cell cultures. Clear overlap was seen with Islet 1 and HB9, confirming the identity of the stained cells. ISL1, Islet 1; HB9, Homeobox domain 9; NfH, Heavy neurofilament.

Immunocytochemistry of iPSC derived MN clearly showed the presence of MN-specific markers Islet 1 and Homeobox gene HB9, as well as the neuron specific marker neurofilament heavy (NEFH). Figure 2 shows the expression of these proteins at two different scales, clearly demonstrating presence and overlap of Islet 1 and HB9 together with NEFH, providing clear evidence of the presence of MN. Control stains with primary antibody only and secondary antibody only showed no fluorescence (data not shown).

### Neuromuscular Junctions Formation Within Microfluidic Chips

**FIGURE 3:**
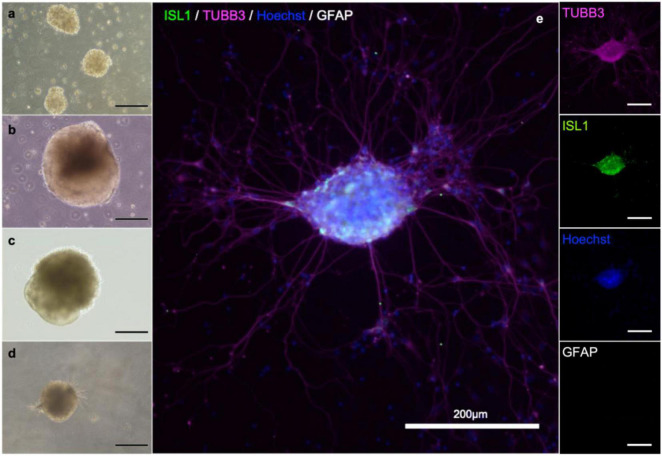
MN aggregate derived from iPS cell reprogramming. Cells during differentiation, corresponding to day 2 **(a)**, 9 **(b)**, 18 **(b)** and 25 **(d)**. After 21 days, MNs are present in the population. Neuronal aggregates **(e)** were formed after dissociation at day 30 and post-seeding and maturation for another 9 days. Immunostaining of the aggregates reveals the presence of immature neurons (β-III-tubulin; magenta), motor neurons (Islet 1; green), and in this batch no astrocytes (GFAP; gray). Nuclear counterstaining with Hoechst (blue).

**FIGURE 4:**
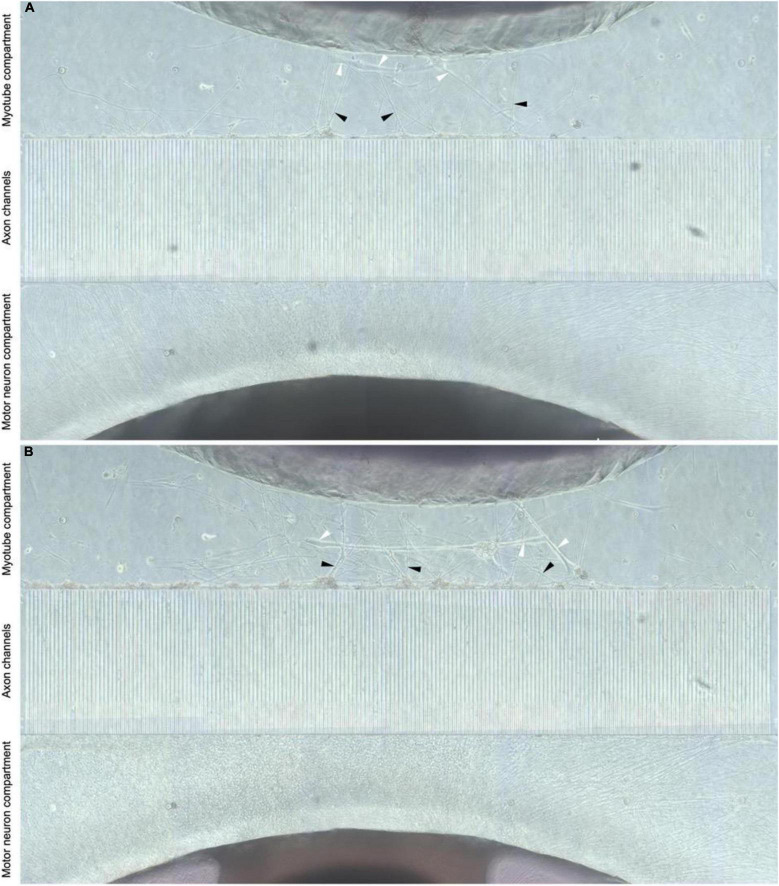
Directional MN axon outgrowth into the myotube compartment. Representative image from the microfluidic chips at 8 and 21 DIV (**A,B**, respectively), showing directional axonal outgrowth from the MN aggregate into the axon tunnels and entry into the opposite compartment containing myotubes. MN neuron axons (black arrows) are clearly visible and appear to contact myotubes (white arrows).

To establish *in vitro* NMJ, pseudo-organoids of the human iPSC derived MNs (Figure 3) and human myotubes were seeded in separate cell compartments in an in-house developed multi-nodal microfluidic chip with directional tunnels connecting the compartment housing the pseudo-organoids with the one containing the myotubes (O’Rourke et al., 2015). MN axon outgrowth began promptly and, typically within 3 days in chips (DIC), the first axons had reached through the tunnels into the adjacent cell compartment (Figure 4). By 8 DIC the first contracting myotubes could be observed. Larger myotubes were also observed developing spontaneous activity without direct contact with MN axons. The diameter and length of the axon bundles exiting the tunnels and the number of myotubes contacted by these axons continued to increase until all compartments were fixed after 21 DIC. Although some cells from the pseudo-organoids settled onto the bottom of the compartment and migrated away, the vast majority of cells remained within the pseudo-organoids.

### Verification of Synaptic Contact Between Motor Neurons and Myotubes

**FIGURE 5:**
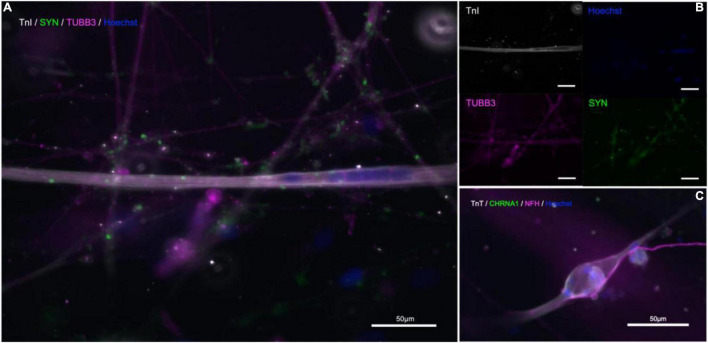
Immunostaining in microfluidic chips indicates presence of NMJs. **(A,B)** Troponin I (TnI, gray), synaptophysin (SYP, green), and β-III-tubulin (TUBB3, magenta), nuclear stain Hoechst (blue). **(C)** Neurofilament-heavy (NF-H, magenta), nicotinic acetylcholine receptor subunit 1 (CHRNA1, green), troponin T (TnT, gray), nuclear stain Hoechst (blue).

**FIGURE 6:**
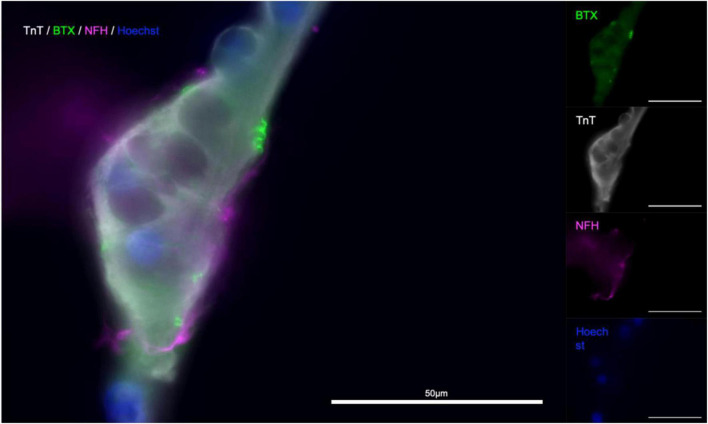
α-Bungarotoxin staining indicates presence of functional NMJs. Troponin T (TnT, gray), α-Bungarotoxin (BTX, green), and Neurofilament-heavy (NF-H, magenta), nuclear stain Hoechst (blue). Merged image (left).

Immunostaining within the microfluidic chips confirmed expression of synaptophysin (SYP), Troponin I (TnI), Troponin T (TnT), Neurofilament heavy (NFH), and beta-III-tubulin (Figure 5). Synaptophysin and CHRNA3 (cholinergic receptor nicotinic alpha 3 subunit) expression could be observed around the surface of the troponin-positive cells (Figure 5), suggesting the presence of NMJs. To confirm that the myotubes in our model were mature, myoblasts were allowed to differentiate under the same media, coating, and media change conditions as within the chips and allowed to differentiate for 21 days. The myotubes that immunostained positive for TnI or TnT and showed the same and similar morphology observed in actively contracting myotubes in the chips also expressed the mature muscle markers CACNA2D1 and Ryanodine receptor (Supplementary Figure 1). Additional immunostaining combined with staining using a fluorescent conjugate of the postsynaptic nicotinic acetylcholine receptor-specific Bungarus multicinctus toxin-derived long-chain α-neurotoxin α-Bungarotoxin (BTX) revealed BTX expression specific to the surface of myotubes that were in contact with axons, as well as expression of skeletal muscle proteins TnI or TnT (Figure 6). Furthermore, most of the myotubes with BTX staining on their surface had been observed contracting before the cultures were fixed. In addition to the above, the presence of mature MNs in the pseudo-organoids was further verified with immunocytochemistry of pseudo-organoid sections revealing cells co-expressing NeuN and HB9 as well as NeuN and Islet1 (Supplementary Figure 2).

### Alpha-Bungarotoxin Abolishes Observable Contractile Activity in the *in vitro* Neuromuscular Junctions

**FIGURE 7:**
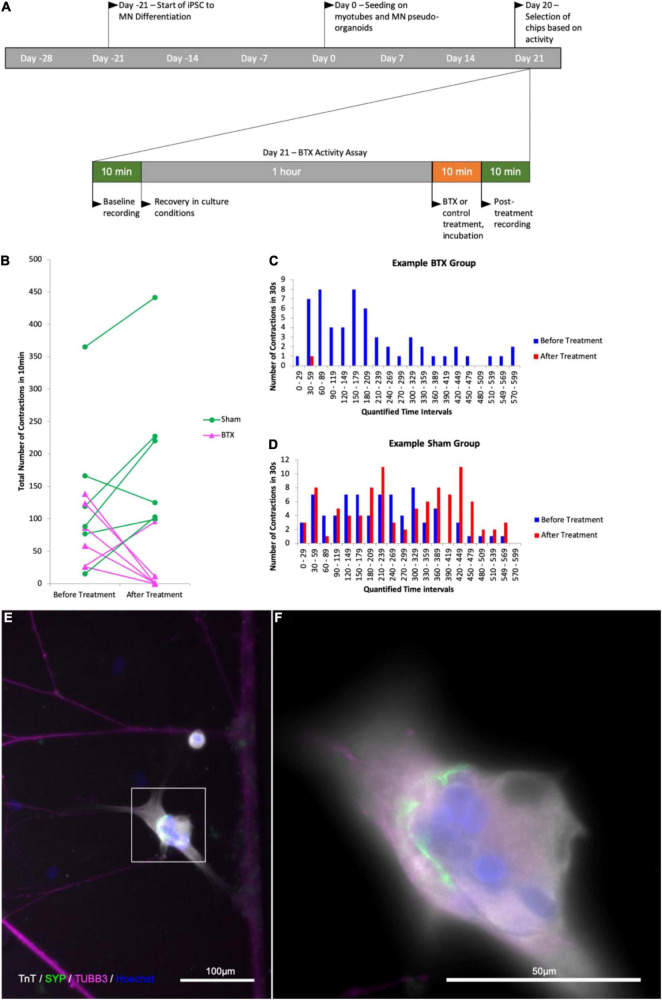
Quantification of contractile activity before and after -Bungarotoxin application BTX binding at myotube surface. Outline of experimental timeline **(A)**, total number of contractions in a 10 min time interval in a myotube in contact with one or more axons in the control and BTX-treated groups before and after treatment **(B)** number of observed myotube contractions in 30 s intervals before and after treatment in a myotube in the BTX-treated **(C)** and sham-treated **(D)** groups. **(E)** Image of myotube and MN axons after BTX application. **(F)** Higher magnification of the myotube in the area boxed in **(E)**; Troponin T (TnT, grays), α-Bungarotoxin (BTX, green), and beta-III-tubulin (magenta), nuclear stain Hoechst (blue).

**FIGURE 8:**
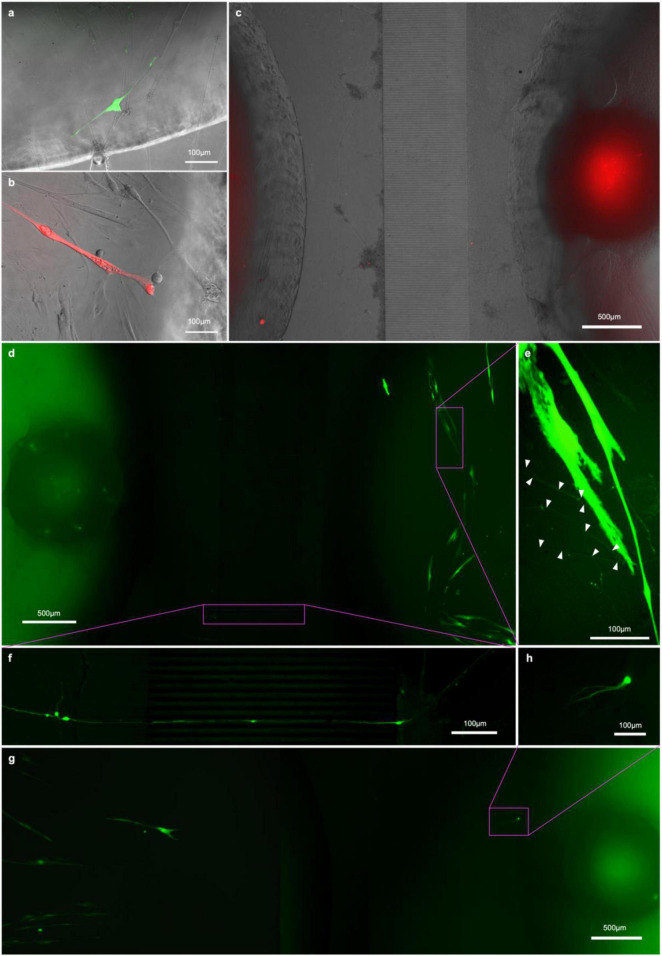
RV-infection and retrograde tracing. **(a)** EnvA-ΔG-RV-GFP and **(B)** EnvA-ΔG-RV-mCherry-infected myotube fluorescence 48 h after infection superimposed on phase-contrast images of the respective myotubes; **(C,D,G)** overview tracing EnvA-ΔG-RV-mCherry fluorescence superimposed on phase-contrast image at 31 DIV **(C)** and EnvA-ΔG-RV-GFP **(D,G)** from infected myotubes to cell bodies inside the aggregates **(D)** and cells that migrated out **(G,H)** at 29 DIV; **(E)** details of axons connecting up the infected myotubes and **(F)** crossing the channels from the outer to the myotube compartment.

To confirm that the contractile activity observed was due to the presence of functional NMJ, we made use of the natural functionality of α-Bungarotoxin, which causes muscle paralysis by blocking activity of the postsynaptic nicotinic acetylcholine receptors in the NMJ. We next wanted to see whether the contractions we were observing in the myotube compartment could be inhibited by introducing α-Bungarotoxin to the cultures. In a pilot experiment, we observed a complete abolition of contractile activity in all experimental group chips, while the control saw a slight increase in contraction frequency. In a blinded follow-up, similar responses were observed within 3 of the myotubes imaged in the experimental group (*n* = 6) having their activity completely abolished, one with a reduction from 58 to 1 contraction in the first minute of the observed window, one with a reduction of contractile frequency by over 91% (124 contractions per 10 min to 11), and the last with short bursts of increased contraction frequency that occurred in groups with an increasing proportion of partial contractions and contractile activity ceasing completely after 6.5 min. Similarly, the myotube that showed a 91% decrease in activity performed one full contraction and 10 partial contractions thereafter (Figure 7). Subsequent imaging of the chips revealed the presence α-Bungarotoxin binding on Tn-positive cells that were previously observed to lose their contractile activity in the experiment (Figure 8), in close proximity to axons (beta-3-tubulin staining).

### Retrograde Tracing With Pseudotyped Rabies Virus Validates Neuromuscular Junctions Functionality

Finally, we wanted to see whether we could demonstrate NMJ functionality by exploiting the natural infection pathway of rabies from muscle to MNs, employing fluorescently labeled, glycoprotein-deleted, pseudotyped rabies virus for monosynaptic retrograde tracing (Wickersham et al., 2010; Osakada and Callaway, 2013).

To this end, we transfected the myoblasts in the central compartment of our chips with helper constructs pCAG-YTB (Marshel et al., 2010) or pAAV-CMV-TVAmCherry-2A-oG (Ciabatti et al., 2017) by electroporation to express the exogenous receptor TVA and rabies-G immediately before seeding them in the compartment. Otherwise, the chips were seeded as before, and the cultures allowed to mature. After 15DIC the cells in the myotube compartment were infected with either EnvA-ΔG-RV-mCherry or EnvA-ΔG-RV-GFP (for pCAG-YTB or pAAV-CMV-TVAmCherry-2A-oG groups, respectively). The rationale for this is that myotubes expressing the helper construct are infected by EnvA-ΔG-RV and subsequently express the transgenes and form virions. Only MNs connected to rabies infected myotubes will receive virions through the synapses. In both experimental groups, the first myotubes showed bright fluorescence by 17 DIV (Figures 8A,B) however, not all transfected myotubes were infected and the myotubes that were infected started becoming less healthy and dying after a few days. By 20 DIC, the first neurons in the pAAV-CMV-TVAmCherry-2A-oG and EnvA-ΔG-RV-GFP group showed observable levels of fluorescence. From 20 DIV until the experiment was terminated at 31 DIV, we observed one or more neurons in at least one pseudo-organoid in each of the pAAV-CMV-TVAmCherry-2A-oG chips becoming infected, with the infected neurons typically dying within a week of becoming bright enough to detect. Somata of the infected neurons were found both amongst the cells that had migrated out from the aggregate (Figure 8H) as well as throughout the aggregate itself (Figure 8D). In the pCAG-YTB group we observed infection of MNs in 2/5 chips, with the first detectable infection only occurring 10 days after initial infection of the myotube compartment.

## Discussion and Conclusion

In this study, we unequivocally establish the functionality of engineered *in vitro* NMJ using recombinant monosynaptic pseudotyped ΔG-rabies virus tracing. To the best of the authors’ knowledge, this is the first study demonstrating validation of *in vitro* NMJ functionality through monosynaptic retrograde tracing with a designer glycoprotein-deleted rabies virus, employing two different helper constructs to allow virus entry to the myotubes and producing infective virus particles. Monosynaptic retrograde tracing, correlated with the abolition of contractile activity in the NMJ using α-BTX, provides unequivocal evidence of the functionality of the NMJs engineered within the microfluid chip.

Having initially verified the antigenic profile of iPSC derived MNs, we proceeded to establish their functionality by assessing the spontaneous activity of MN networks on MEAs over time. Key parameters such as firing rate, together with the bursting behavior, provide a good overall impression of the state of the network, with regard to emergence of spontaneous electrical activity and synchronization, respectively. Identification of three distinct stages of MN network maturity suggests that early increases in overall activity in the first 5 weeks does not indicate the establishment of a well-connected network, as significant bursting activity is not observed until 42 DIV, at which point the firing rate is already quite high and does not significantly increase further. Following this, even though overall activity decreases, as seen in the firing rate, the activity remained highly synchronized and mostly contained in burst firing, consistent with previous findings (Wagenaar et al., 2005). However, it appears that networks of motor neurons mature into synchronized states much slower than what is previously reported with cortical neurons (Cotterill et al., 2016). Taken together, these results are important as they show that the iPSC derived MNs form functional, coordinated networks before proceeding with the *in vitro* NMJ assays.

We chose to deliver the TVA and rabies glycoprotein-containing single helper constructs, required to allow infection of the myoblasts by the pseudotyped ΔG-rabies virus and production of infectious viral particles, respectively, by electroporation over other delivery methods. Only the myotubes expressing the helper construct were infected upon application of EnvA-ΔG-RV (subsequently expressing transgenes and forming virions). In turn, only MNs synaptically connected with the rabies expressing myotubes received virions through the synaptic contact between MNs and infected myotubes. Virus expressing MNs were unable to form infectious virus particles due to lack of rabies-G, hence the viruses could not propagate further from one infected MN to another. This approach avoided any risk of chance infection of one of the axons extending into the myotube chamber of our microfluidic chips that would have been theoretically possible had the helper construct been administered *via* virus. The lower infection efficiency in the group using pCAG-YTB as a vector for myotube infection was expected, as the more recently developed pAAV-CMV-TVAmCherry-2A-oG contained a chimeric codon-optimized version of rabies-G that showed up to 20 times higher tracing efficiency in mouse models (Kim et al., 2016). That being said, should ΔG-rabies tracing become more common practice in human *in vitro* network and/or disease models, there may be room for screening through and codon optimizing chimeric rabies-Gs specifically for improved tracing efficiency in human cell culture. Notwithstanding the above, we saw transmission of the virus from the infected myotubes to MNs with both constructs. This supports the notion that our model provides a robust platform for investigating mechanisms involving NMJ formation and function. It is noteworthy that, while there were some infected MNs that had migrated out of the MN aggregates (presumably after having established contact with the myotubes), most of the infected cells were located inside the pseudo-organoids. Interestingly, some of the cell bodies that showed fluorescence were located on the far side of the MN aggregates making visual detection challenging.

We repeatedly observed that RV-infected myotubes contacted by one or more axons would become brightly fluorescent, detach and die in the days before the associated axons and respective cell bodies became fluorescent enough to visualize and trace. Given the brightness of the fluorescence usually observed before this occurred, it was not unexpected, as it correlates with the extent to which the cellular machinery is taken over by the virus for production of more viral particles and the corresponding load on homeostatic mechanisms (Ciabatti et al., 2017).

α-BTX has been shown to have a similarly high affinity to the neuronal nACHRs subunits α7 and α9/10 (Couturier et al., 1990). The ease of controlling for this in our microfluidic platform of choice, by ensuring unidirectional flow of media from the outer to the inner compartment through hydrostatic pressure, demonstrates the practical advantage of this feature of the microfluidic platform.

The addition of α-BTX completely abolished contractile activity in the *in vitro* NMJs, except in one unit, in which some contractile activity could still be observed. The latter was not entirely surprising, given that cultured myotubes tend to show spontaneous activity in culture (Guo et al., 2014; Al Samid et al., 2018). It was particularly of note that the kind of contraction observed changed in the myotubes that did not show complete abolishment of activity. In one case the activity was reduced to one full contraction and ten partial contractions in two groups as opposed to a mix of both occurring throughout the observed period before addition of α-BTX. This incomplete or delayed effect may have been due to a delay in mixing speed between bulk media in the compartment and the narrow space within the active zone of the microfluidic chip, which, in some cases may have been exacerbated by the presence of large axon bundles around the myotube.

Taken together, the above experiments showed that the NMJs formed were functional both in their postsynaptic acetylcholine-dependent induced contractions, as well as in key presynaptic vesicle formation and retrograde transport mechanisms that RV relies on for infecting neurons.

Preclinical *in vivo* models largely involve the generation of transgenic mice overexpressing some of the most common gene mutations associated with ALS. For example, various transgenic mutant mouse lines overexpressing the SOD1 mutation have been generated. These mutants recapitulate hallmarks of ALS pathology, such as limb tremor, locomotor deficits, and paralysis (Nardo et al., 2016). Similarly, the TDP-43 mutant mouse models display positive cytoplasmic inclusions of the protein (Kabashi et al., 2008), while FUS models manifest progressive, mutant-dependent MN degeneration (Philips and Rothstein, 2015) and functional abnormalities in the NMJ (Sharma et al., 2016). On the other hand, while C9ORF72 mutants may display evidence of ALS-related pathology, such as a microglia phenotype or frontotemporal dementia (FTD), they fail to recapitulate MN degeneration (Philips and Rothstein, 2015). Thus, while the SOD1, TDP-43, FUS, and C90RF72 mouse mutant lines may afford highly useful insights into specific pathological features and disease mechanisms of ALS, no single model can fully capture the immense complexity of the disease or faithfully mimic the human patient organism. Similar limitations apply to other vertebrate, as well as invertebrate models of ALS (Matthaei, 2007; Watson et al., 2008; Perrin, 2014).

Considering the above, *in vitro* models, including NMJ engineering approaches are highly relevant for studying and elucidating mechanistic causes of the disease. In recent years there has been an increasing interest in high quality *in vitro* models to examine the NMJ, which poses unique challenges in terms of co-culturing cells with different environmental and nutritional requirements (Natarajan et al., 2019). The use of microfluidic devices and human iPS cells represents a promising avenue for investigating healthy function of the specialized NMJ structure, as well as model CNS injury and disease. In neurodegenerative diseases such as ALS, microfluidic models have shown promise in the investigation of the effectiveness of drugs delivered to the distal axons located outside the CNS as opposed to delivery to the soma, which is more difficult to target with treatment *in vivo* (Marshall and Farah, 2021). Microfluidic models using ALS patient specific iPSC derived MNs expressing mutant FUS demonstrated a reduction in neurite outgrowth and the number of total NMJs and showed that histone deacetylase 6 inhibition could rescue outgrowth impairments and improve NMJ formation (Stoklund Dittlau et al., 2021). Another study using a 3D microfluidic approach demonstrated that motor units using iPSC derived motor neurons from a patient with sporadic ALS produced weaker muscle cell contractions compared to healthy donor derived controls, and treatment with mTOR pathway inhibitors increased muscle contractile force and improved motor neuron survival in the ALS model (Osaki et al., 2018). The potential incorporation of different cell types, such as various glial cells, can further improve the relevance of these models to the specific conditions of ALS or other diseases where the NMJ is selectively vulnerable (de Jongh et al., 2021; Arjmand et al., 2022). Using an in-house developed microfluidic chip (van de Wijdeven et al., 2018, 2019), we have been able to reproducibly generate functional NMJs from human iPSC derived MNs and primary myotubes. The specific microfluidic platform offers a particular advantage to other previously described approaches in that the small axon channels with controllable connectivity allow for near complete isolation of extracellular environments through use of hydrostatic pressure, thus enabling chemical insult to NMJs and MN cell bodies to be carried out independently (van de Wijdeven et al., 2018, 2019). By the same token, this differentially perturbable microfluidic environment enabled selective application and functional retrograde tracing of the recombinant monosynaptic pseudotyped ΔG-rabies virus from the NMJ to the MN soma for unequivocal validation of NMJ functionality. As discussed above, the specific approach is particularly relevant for *in vitro* ALS disease modeling as it provides an elegant way of testing the dying back hypothesis with regard to the initiation and spread of the ALS pathological cascade. Conversely, designer viral tools for anterograde monosynaptic tracing can be selectively applied to MN for the study of NMJ responses, in line with the dying forward hypothesis. Thus, designer viral tools for selective manipulation of muscles, neurons, and synapses, combined with microfluidic systems, including microfluidic MEA interfaces that enable electrophysiological recordings, hold great promise for preclinica*l in vitro* modeling of ALS, including ALS patient-specific modeling, including preliminary drug screening. Such *in vitro* approaches are thus highly complementary to *in vivo* disease modeling, while they also have the potential of reducing the need for animal models.

## Data Availability Statement

The raw data supporting the conclusions of this article will be made available by the authors, without undue reservation.

## Author Contributions

UB, AS, and IS conceptualized the study. RN and CK developed designer viral tools. RW designed and produced the microfluidic platform. UB, VF, and RW performed the experiments and analysis. UB, VF, AS, and IS wrote the manuscript. All authors edited and finalized the manuscript.

## Conflict of Interest

The authors declare that the research was conducted in the absence of any commercial or financial relationships that could be construed as a potential conflict of interest.

## Publisher’s Note

All claims expressed in this article are solely those of the authors and do not necessarily represent those of their affiliated organizations, or those of the publisher, the editors and the reviewers. Any product that may be evaluated in this article, or claim that may be made by its manufacturer, is not guaranteed or endorsed by the publisher.

## Footnotes

1
https://www.addgene.org/104330/

2
https://www.addgene.org/26721/
