# Supplementary material for: Validation of Functional Connectivity of Engineered Neuromuscular Junction With Recombinant Monosynaptic Pseudotyped ΔG-Rabies Virus Tracing
Source: Front Integr Neurosci. 2022 May 20;16:855071. doi: 10.3389/fnint.2022.855071 (PMC9163662; doi:10.3389/fnint.2022.855071)
Supplement: Supplementary file 1 [file Image_1.pdf]

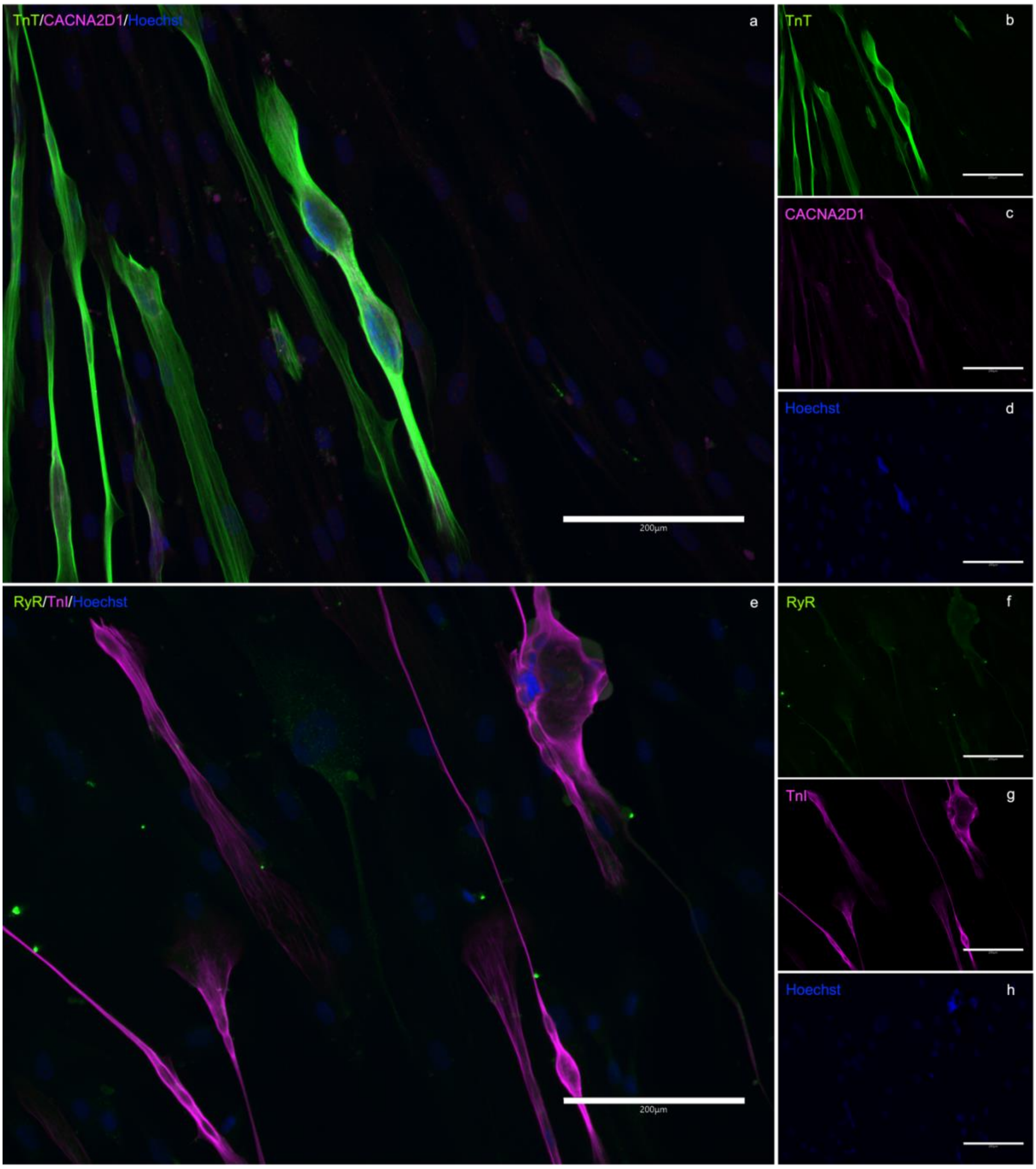

Supplementary Figure 1. Coexpression of Troponins I and T with Mature Muscle Markers
Myotubes displaying morphology suggestive of contractive activity after 21DIV of differentiation showed (a) coexpression of Troponin T (green – b) with Calcium channel L type DHPR alpha 2 subunit/CACNA2D1 (magenta – c) when immunostained, nuclear staining with Hoechst (blue – d), as well as (e) coexpression of the Ryanodine Receptor (green – f) with Troponin I (magenta – g), nuclear staining with Hoechst (blue – h).

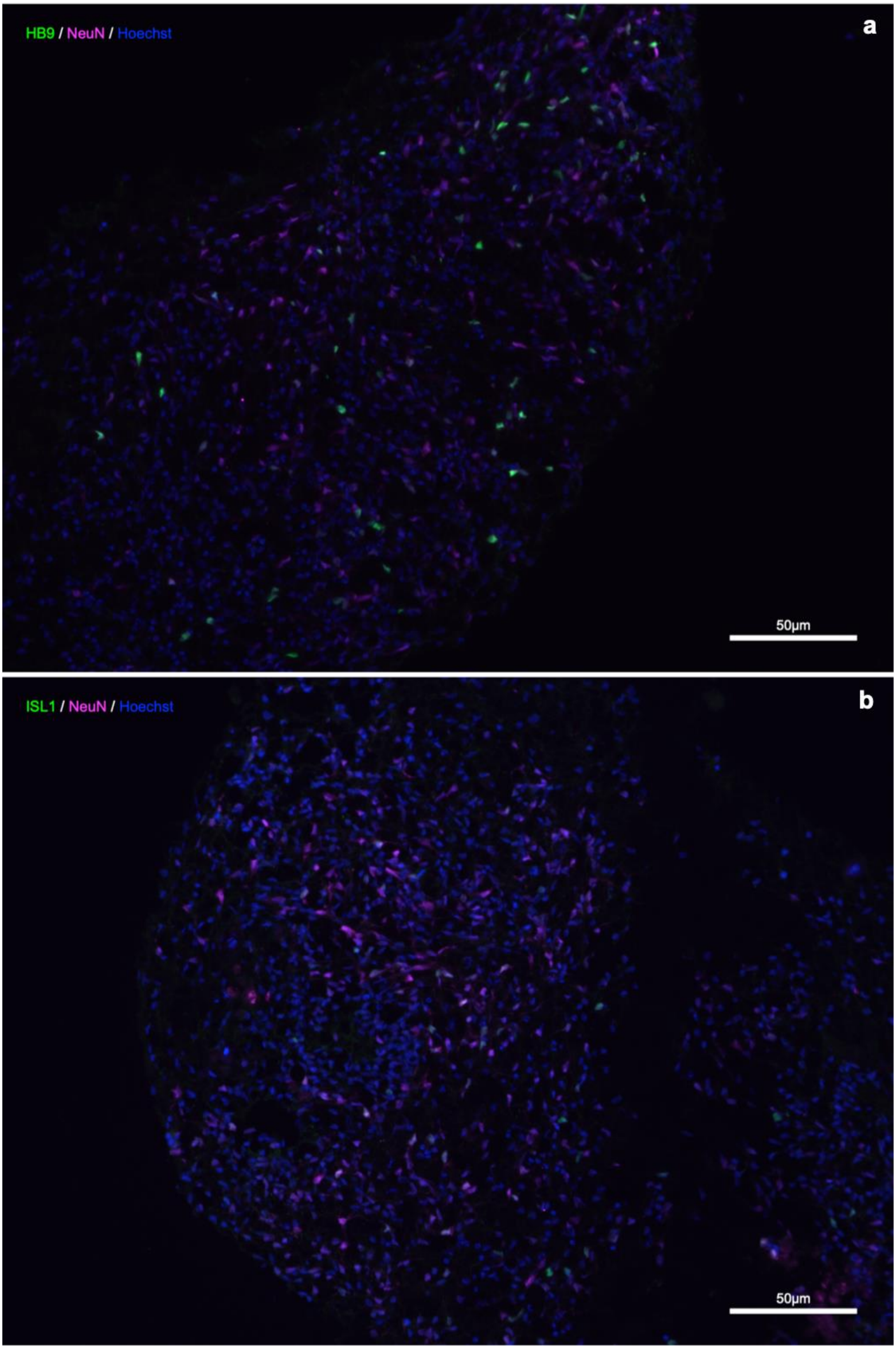

Supplementary Figure 2. HB9 and ISL1 expression in Sections of MN aggregates.
Sections of MN aggregates immunolabelled for NeuN (magenta) and (**a**) Homeobox HB9 (HB9, green) or Islet 1 (ISL1, green), nuclear staining with Hoechst (Blue)
